# Constant dedication, passion and devotion for the patients with neurological affections

**Published:** 2015

**Authors:** VL Purcarea

**Affiliations:** * “Carol Davila” University of Medicine and Pharmacy, Bucharest, Romania

In less than one year, the dream of the Foundation for the Study of Nanoneuroscience and Neuroregeneration, ***RoNeuro Institute***, an academic excellence research and diagnosis center for the neurological diseases, came true, managing to treat over 3000 patients, most of them representing difficult cases in Romania and abroad. The existence of not only some remarkable professionals but also the latest technologies in the field, which were used for the first time in Romania, as for example the eye-tracking system, in the process of evaluating the cognitive performances, has made possible its fully deserved assertion in the elite medical world. 

Organized by the ***Society for the Study of Neuroprotection and Neuroplasticity*** (SSNN), together with the ***Romanian Society of Neurology*** (RSN) and ***“Iuliu Haţieganu” University of Medicine and Pharmacy*** in Cluj-Napoca, under the aegis of the ***European Federation of NeuroRehabilitation Societies*** (EFNRS) and the **World Federation for NeuroRehabilitation **(WFNR), the scientific and exceptional educational medical event, ***RoNeuro Brain Days***, which hosted the 5th edition of the **European Teaching Course on NeuroRehabilitation**, and which took place in the period 1-4 of June 2015, in ***Cluj-Napoca***, the location being “Gheorghe Marinescu” Amphitheatre in “Iuliu Haţieganu” University of Medicine and Pharmacy, has managed to bring together professionals in the field of medicine and world scientific researches, from countries such as ***Germany, USA, Austria, Italy, Ukraine and Romania***.

**Fig. 1:**
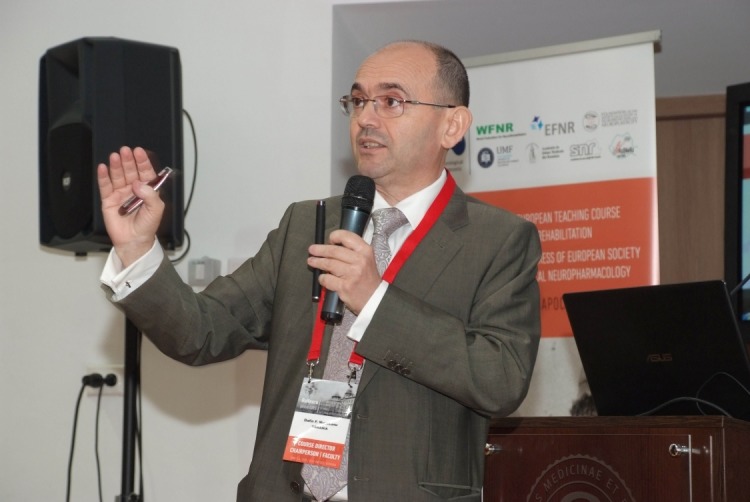
Prof. Dafin F. Mureşanu, MD, President of the Romanian Society of Neurology (RSN) and President of the Society for the Study of Neuroprotection and Neuroplasticity (SSNN)

*“We would like to develop rehabilitation in Romania in a modern, interdisciplinary context, in which the neurologist, the specialist in physical medicine and recovery medicine, the psychologist, logopedist, kinesiotherapist, ergotherapist and medical assistant work together as a team and generate the optimum result for the patient. We would also try to bring a neurorehabilitation component in the field, in the near future, a curriculum being already developed at the European level. Neurorehabilitation represents an interdisciplinary growing field in Europe. Many national societies are born under our sight, but certainly, the leaders are still the national societies in Germany, Italy, Holland, Austria. Romania is active in this field with the help of the two neurorehabilitation societies”, **Prof. Dafin F. Mureşanu, MD**, President of the Romanian Society of Neurology (RSN) and President of the Society for the Study of Neuroprotection and Neuroplasticity (SSNN), affirmed.*

**Fig. 2:**
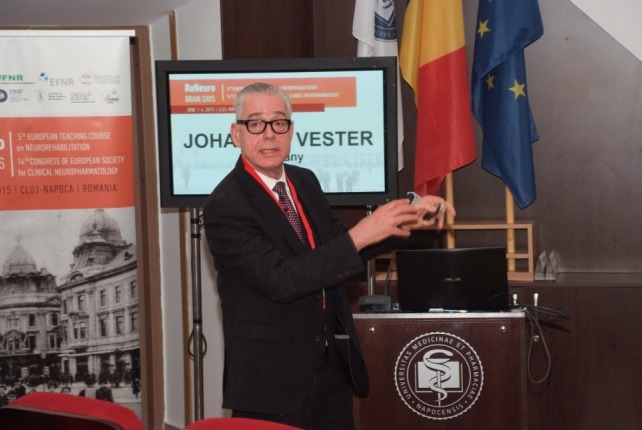
Prof. Johannes Vester, MD, Germany Senior Consultant Biometry and Clinical Research IDV - Data Analysis and Study Planning, Germany

**Fig. 3:**
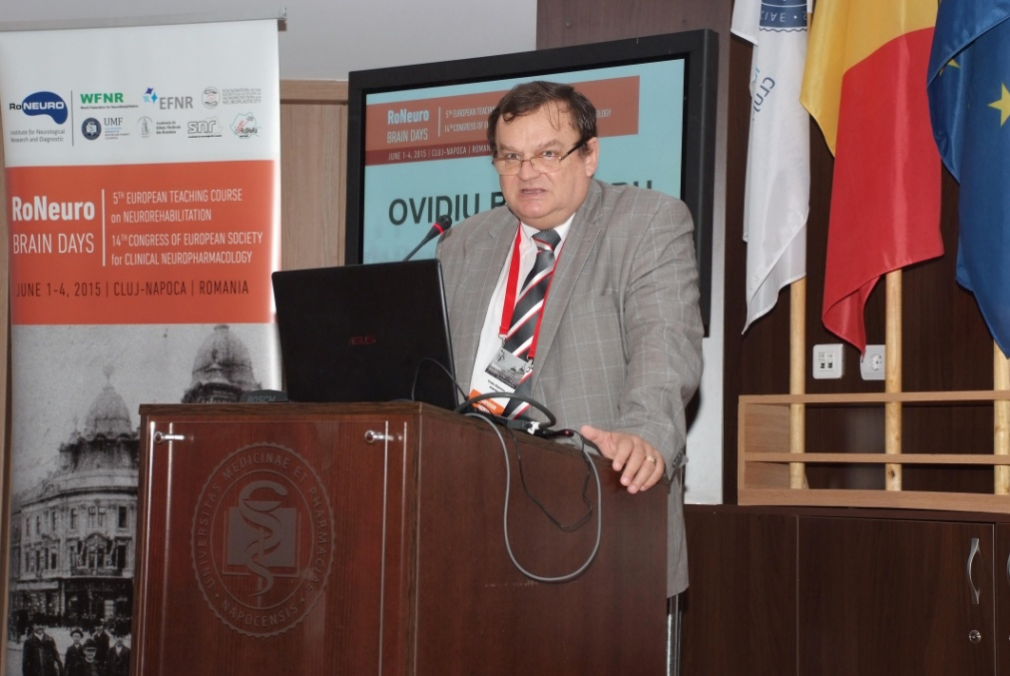
Prof. Ovidiu Băjenaru, MD, Romania “Carol Davila” University of Medicine and Pharmacy, Bucharest, Romania Director of the Department of Neurology, Neurosurgery and Psychiatry Chairman and Head of Dept. Neurology - University Emergency Hospital, Bucharest, Romania

Premature brain aging and brain regeneration have been the main preoccupations of the elite of neurologists who took part in the event in Cluj. *“There are some factors and the lifestyle is the most important. Hypertension, atrial fibrillation, dyslipidemia, diabetes, physical inactivity, obesity, smoking, exaggerated alcohol or central nervous system stimulant substances consumption, intellectual overstrain, stress, hormonal dysfunctions, brain trauma, are reasons which can generate an early brain aging”*, **Prof. Dafin F. Mureşanu, MD**, declared. 

**Fig. 4:**
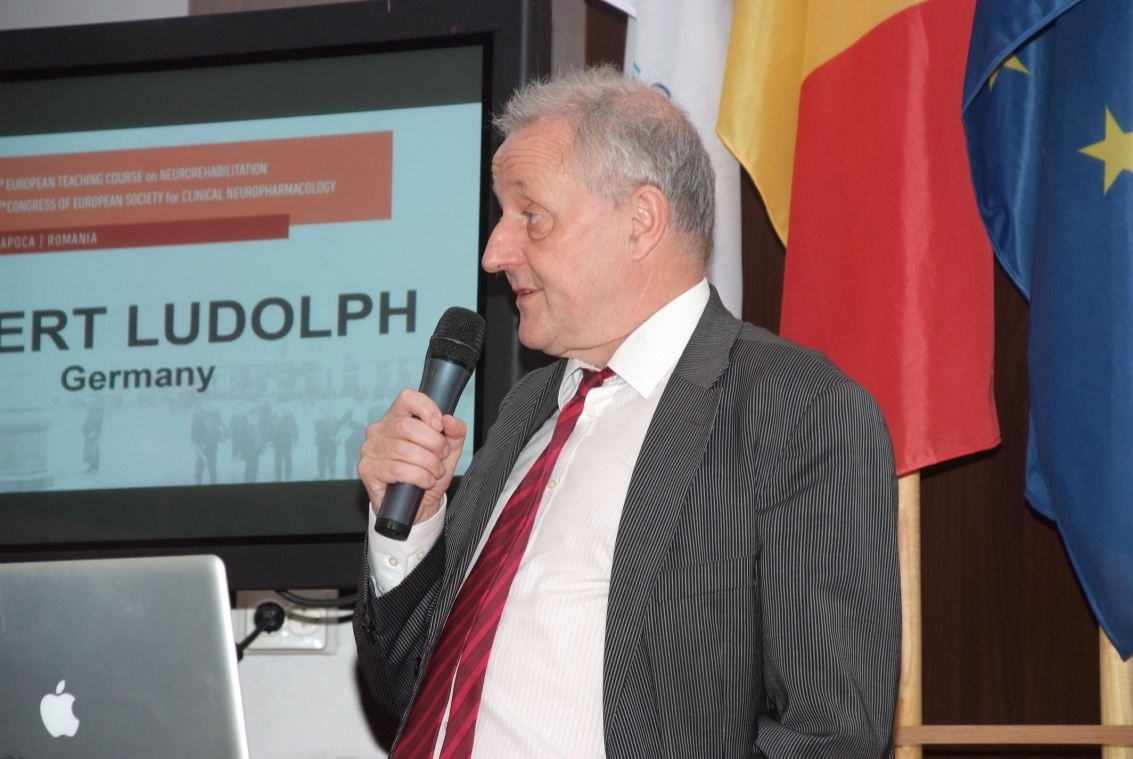
Prof. Albert Ludolph, MD, University of Ulm, Department of Neurology; Medical director, Head of Department, Ulm, Germany

**Fig. 5:**
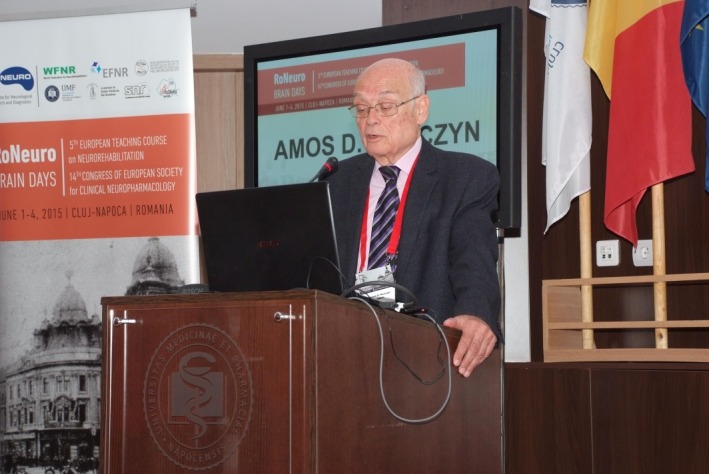
Amos D. Korczyn, Israel, Sackler School of Medicine, Tel-Aviv University, Israel

The greatest retrospective traumatic brain study in the last decade has been presented in **RoNeuro Brain Days**, which drew the attention to the positive effects of the treatment with neurotrophic factors in patients with this type of brain injuries. According to these data, the treatment significantly improves the patients’ quality of life. In this complex study, coordinated by Prof. Dafin F. Mureşanu, 25 top neurosurgeons and neurologists have taken part, mostly from Romania, who have treated and monitored 7.769 patients on a period of 5 years, in 10 neurosurgery departments in Romania. 

*“This study has been a real challenge for neurology in general and especially for neurosurgery. The results have shown an obvious improvement of the health state of the patients who suffer from brain injuries, which are mostly produced due to road accidents, to which the new treatment had been applied in the first 48 hours from the accident”*, Prof. Fafin F. Mureşanu, MD, commented.

During **RoNeuro Brain Days**, another event took place, **The Congress of the European Society for Clinical Neuropharmacology** (ESCNP), which is one of the elite societies of world medicine, whose president elected last year, is **Prof. Dafin F. Mureşanu, MD**. In addition, **Prof. Mureşanu** continues the projects of his predecessor, Prof. Johannes Thome, MD, from Germany, a scientific society whose purpose is the promotion of education and acquiring a high level of knowledge and understanding in the field of clinical neuropharmacology. The society supports the research activity in neuropharmacology, the development of scientific standards, counseling regarding the best methods and techniques to interpret the results but also maintaining the collaboration with the national and international societies, governmental organizations, professional associations, as well as other types of groups, societies, institutions, etc., which contribute in the development of the clinical neuropharmacology field. 

**Fig. 6:**
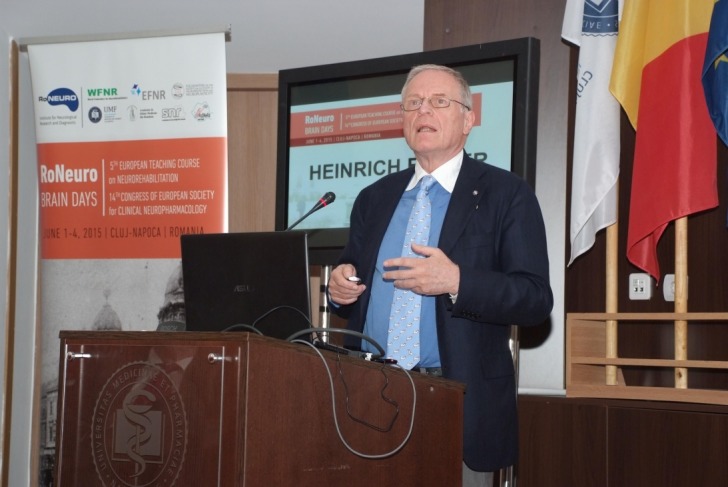
Prof. Dr. Heinrich Binder, MD, Landsteiner Institute for Neurorehabilitation and Space Medicine Vienna, Austria, President of Austrian Society for Neurorehabilitation (OEGNR), President of the European Federation NeuroRehabilitation Societies (EFNRS), Member of Management Committee of the World Federation NeuroRehabilitation (WFNR), Chairman of Special Interest Group/WFNR “Spinal Cord Injury”

**Fig. 7:**
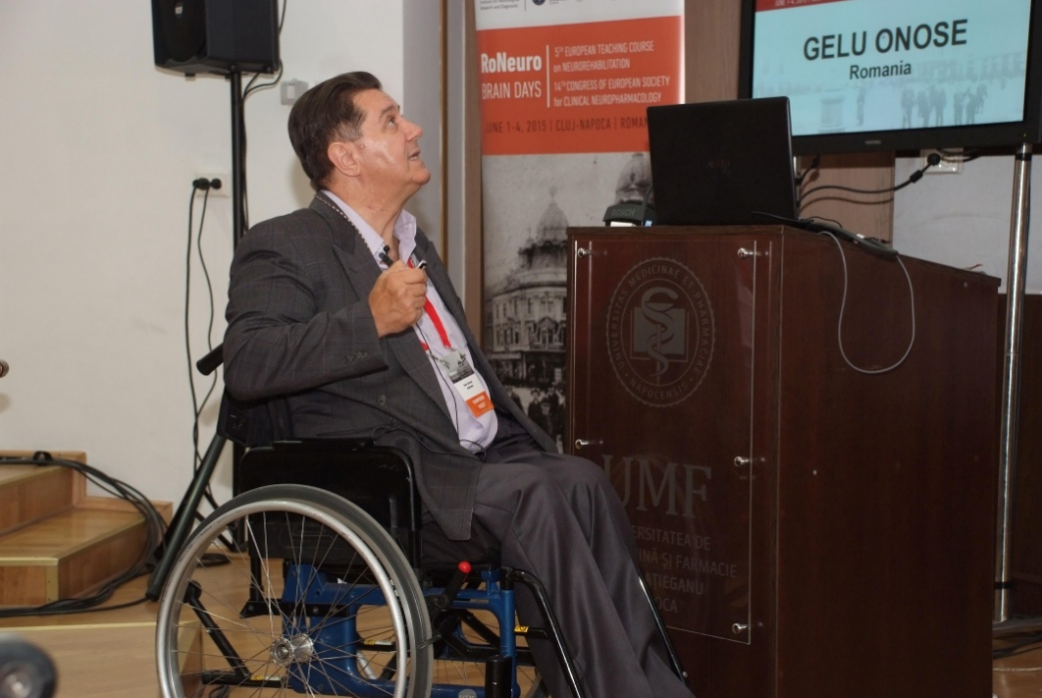
Prof. Gelu Onose, MD, Romania, President Co-Founder of the Romanian Society for Neurorehabilitation (RoSNeRa); Chief of the of the UMPCD PRM Discipline and of the P(neural-muscular)RM Clinic Division - the National Reference Center for NeuroRehabilitation - and of its RDI Nucleus, at “Bagdasar Arseni” Teaching Emergency Hospital (BATEH) in Bucharest

During the event, the following works and papers were presented: Prof. Dafin F. Mureşanu, MD, Romania - Advances in neurorehabilitation fundamentals - an update, Results from a large retrospective cohort trial in TBI, Pharmacological support in traumatic brain and spinal cord injury, The role of neurotrophic factors in brain protection and recovery after stroke; Prof. Albert Ludolph, MD, Germany - ALS genotypes and phenotypes; Prof. Heinrich Binder, MD, Vienna, Austria - The forgotten autonomous system in early rehabilitation, What’s the meaning of early rehabilitation in neurodegenerative diseases?; Prof. Ovidiu Băjenaru, MD, Romania - Neurorehabilitation strategy in patients with focal dystonia, Brain cholesterol: implications in the treatment of neurological diseases; Prof. Amos D. Korczyn, MD, Israel – Disease course modification in Parkinson’s disease, Vascular Parkinsonism, Medically unexplained symptoms in neurology.

**Fig. 8:**
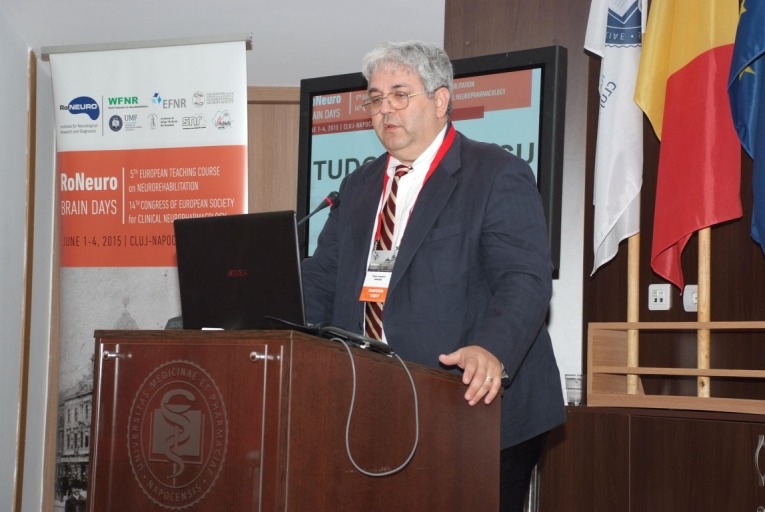
Prof. Tudor Lupescu, MD, Romania, Head of Department of Neurology of “Agrippa Ionescu” Hospital, Bucharest; President of ASNER – Romanian Society of Electrodiagnostic Neurophysiology, Vice-president of the Romanian Society of Diabetic Neuropathy

**Fig. 9:**
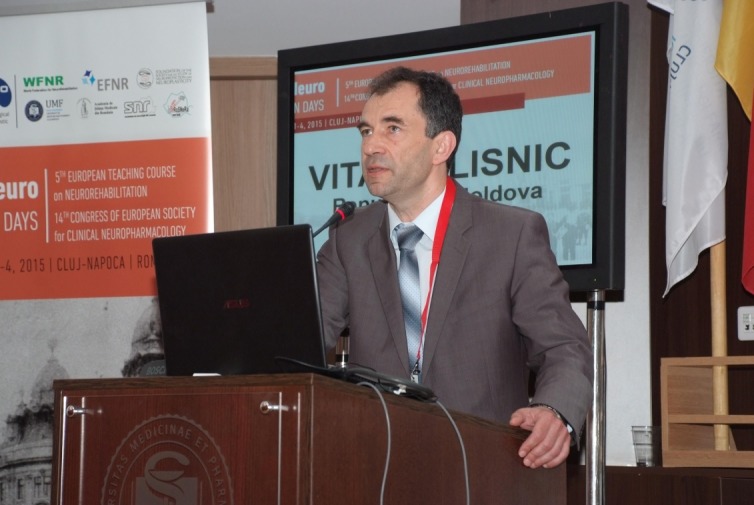
Prof. Vitalie Lisnic, MD, Republic of Moldova, “Nicolae Testemiţanu” University of Medicine and Pharmacy, Chisinau, Republic of Moldova

Moreover, other works were also presented: Prof. Johannes Vester, MD, Germany – Is there a chance for clinical research in neurorehabilitation within the framework of evidenced-based medicine? Classic and new approaches; Prof. Gelu Onose, MD, Romania – Propaedeutics for rehabilitation in the central nervous system traumatology (postacute/ subchronic stages); Prof. Adriana Sarah Nica, MD, Romania – Nutritional care of neurological disabled patients; Prof. Mihaela Baciuţ, MD, Romania - The benefit of high-end neurological therapy in maxillofacial surgery; Prof. Tudor Lupescu, MD, Romania – Symptomatic treatment in diabetic neuropathy; Prof. Vitalie Lisnic, MD, Republica Moldova - Impairment of the central nervous system in demyelinating polyneuropathies: neurophysiological, clinical and neuroimaging aspects, Atypical forms of chronic inflammatory demyelinating polyneuropathy, etc. 

**Fig. 10:**
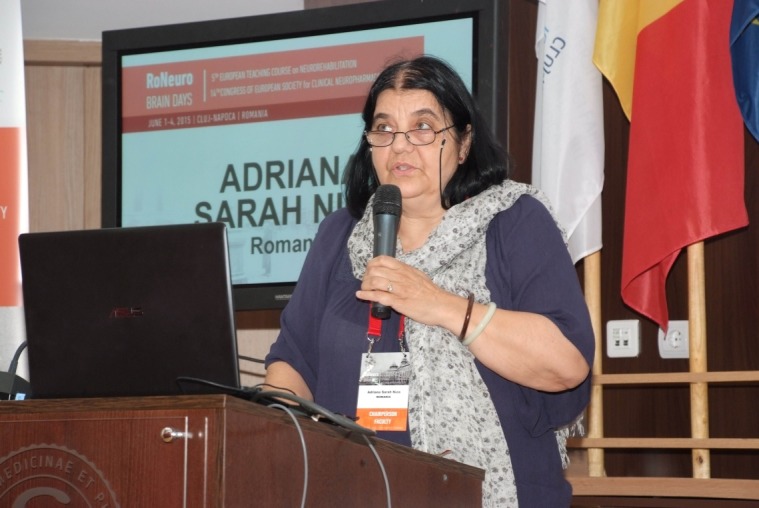
Prof. Adriana Sarah Nica, MD, Romania, Professor in Physical Medicine, Rehabilitation and Balneoclimatology “Carol Davila” University of Medicine and Pharmacy, Bucharest; Head of Rehabilitation Department – “Carol Davila” University of Medicine, Bucharest; Chief of University Rehabilitation Department III – National Institute of Rehabilitation, Physical Medicine and Balneoclimatology

**Fig. 11:**
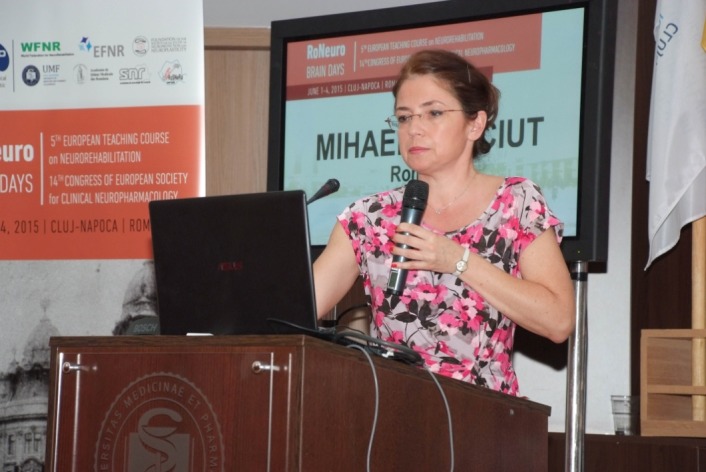
Prof. Mihaela Baciuţ, MD, Romania; Professor, Department of Maxillofacial Surgery and Implantology, Faculty of Dental Medicine, University of Medicine and Pharmacy Cluj-Napoca; Founding member of the Romanian Society of Reconstructive Microsurgery; Vice-president of the Romanian Society of Oral and Maxillofacial Surgery (SRCOMF)

What has already become a tradition is the care of the organizers for the variety and diversity of the actions auxiliary to the scientific ones, which have also been surprising on this event. And, it could not have been otherwise, since it was for example a moment, which was aptly chosen from the works of some illustrious classical music composers, such as Verdi, Rossini, Leoncavallo, Bizet, Offenbach, Lehar, from a bunch of immortal canzonets or traditional Russian or Jewish music, a moment which entirely belonged to the members of the National Opera in Cluj, who are young but very talented, impressing and completing the exceptional scientific congress. 

**Figure:**
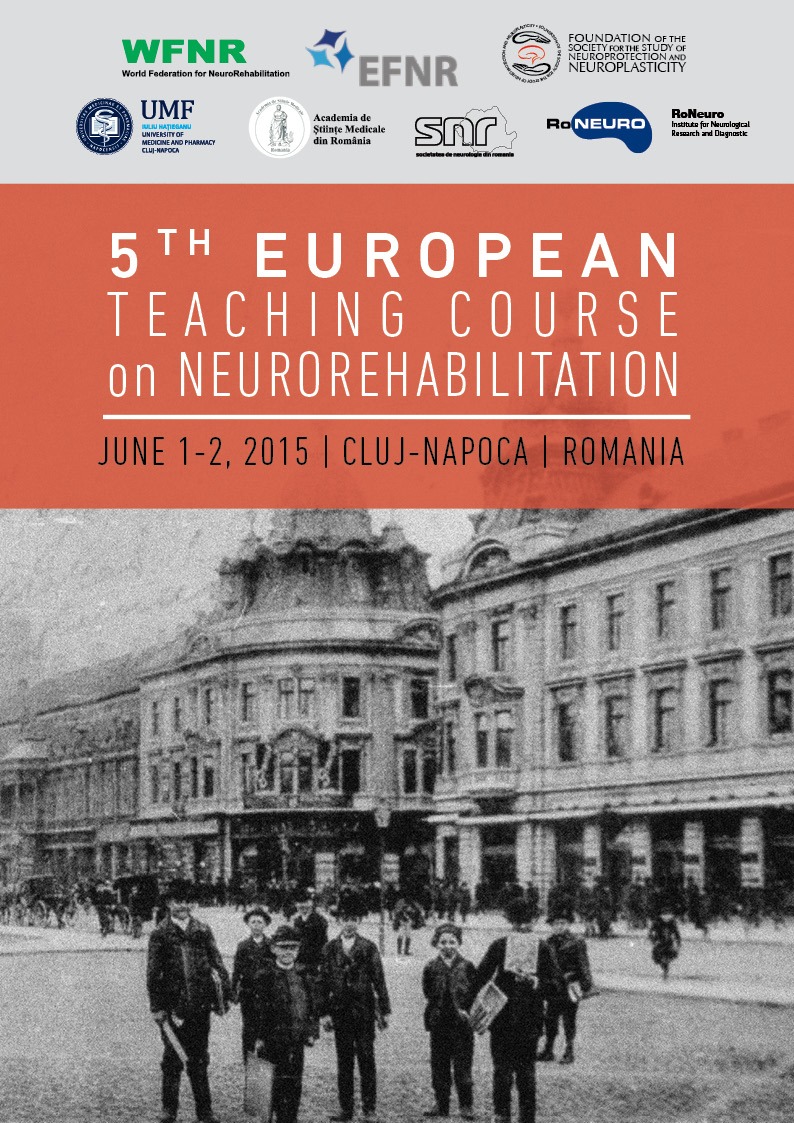


**Executive Editor** **Assoc. Prof. Dr. Eng. Victor Lorin Purcarea**

